# Nurses' perception of solutions proposed by nurse leaders in face of COVID‐19 pandemic: A cross‐sectional study

**DOI:** 10.1111/jonm.13896

**Published:** 2022-11-18

**Authors:** Juliana Santana de Freitas, Roberta Maria Savieto, Ana Laura Queiroz Melo, Isabelle Maria Bortotti, Cláudia Regina Laselva, Eliseth Ribeiro Leão

**Affiliations:** ^1^ Hospital Israelita Albert Einstein São Paulo Brazil

**Keywords:** COVID‐19, healthcare, hospital personnel administration, leadership, nursing

## Abstract

**Aim:**

To evaluate the perception of bedside nurses regarding the implementation of solutions proposed by nurse leaders for patient and employee care during the COVID‐19 pandemic.

**Background:**

Nurse leaders have proposed solutions to better manage the challenges of the pandemic. However, multiple factors influence the transposal of actions from the tactical to the operational levels.

**Method:**

This cross‐sectional study was carried out in a 620‐bed non‐profit institution. Participants were bedside nurses who completed an online survey.

**Results:**

One hundred sixty‐eight nurses participated in the study. Most of the proposed solutions were very effective and easily identified by the nurses. These solutions included adaptations of the physical structure, availability of medical supplies and adequacy of institutional protocols. The actions that stood out with low perception were adequate integration of new employees and the availability of remote work, hotel accommodations for frontline health care workers and day care for children whose parents worked at the hospital.

**Conclusion:**

Bedside nurses were able to recognize most of the solutions proposed by their nurse leaders during the COVID‐19 pandemic.

**Implications for nursing management:**

Tactical‐level nurse leaders need constant proximity to bedside nurses and continuous elucidation of the objectives to be achieved by the strategies adopted.

## 1 BACKGROUND

The first case of infection by the SARS‐CoV‐2 coronavirus was identified in China in December 2019. Since then, infections have spread rapidly and have already reached the six continents. In March 2020, COVID‐19 was considered a pandemic by the World Health Organization (WHO) (Guo et al., 2020). A global response from health systems has been observed, together with a growing concern with the ability to effectively respond to the needs of patients (Remuzzi & Remuzzi, 2020).

During the COVID‐19 pandemic, the WHO outlined a four‐pronged strategy: (1) prepare and be ready; (2) detect, prevent and treat; (3) reduce and suppress and (4) innovate and improve (WHO, 2020). In all instances, nurses play a fundamental role (Newby et al., 2020).

Historically, nurses have played essential roles in major crises humanity faced, such as wars, catastrophes, epidemics and pandemics. In this context, the nurse Florence Nightingale stands out. By implementing handwashing and other hygiene practices, she effectively reduced the number of infections at her hospital during the Crimean war (Loveday, 2020). Besides being a daily practice in hospitals, handwashing has occupied a prominent place in COVID‐19 prevention and has been recommended to the general population.

Nursing professionals who occupy bedside care positions are essential in the collective efforts to prevent and respond to COVID‐19. Nurses provide frontline care for most patients, including complex cases that require hospitalization. Nursing resources are also critical to managing individuals with pre‐existing health vulnerabilities and thus are at high risk of complications or mortality from COVID‐19 (Choi et al., 2020).

Nurses also play a central role in providing public education on disease prevention and reducing the spread of misinformation during the outbreak (Choi et al., 2020). However, for nursing care to be efficient, the articulated work of nurse leaders needs to align with the institutional mission, vision and values; the recommendations of national and international regulatory bodies and the scientific evidence.

This work was based on a previous assessment study with nurse leaders (Freitas et al., 2021). Nurse leaders have proposed solutions for the best management of the challenges posed by the pandemic on health care. The designed solutions, applied by the bedside nurses, resulted in qualified care compatible with worldwide recommendations and, consequently, in better patient experience.

Transposing strategies from the tactical to the operational level is influenced by multiple factors. Thus, knowing the diffusion of those strategies on the frontline is crucial for organizational self‐analysis. Similarly, knowing how the plan was perceived is crucial for adaptation to similar situations, considering that the pandemic persists in Brazil and other locations worldwide.

In this context, we decided to develop this study, which aimed to evaluate the perception of bedside nurses regarding the implementation of solutions proposed by their nurse leaders for patient and employee care during the COVID‐19 pandemic.

## 2 MATERIALS AND METHODS

### 2.1 Design and setting

This is an exploratory cross‐sectional study, whose data were collected in July 2020 at Hospital Israelita Albert Einstein (HIAE) in São Paulo, Brazil. HIAE is a 620‐bed non‐profit institution dedicated to supplementary health care, considered the national reference for COVID‐19, in which the first Brazilian case of the disease was identified.

The hospital is a Joint Commission International (JCI) accredited organization since 1999 and a Planetree‐certified hospital since 2011 and, at the time of data collection for this work, was on the journey to achieve the American Nurses Credentialing Center (ANCC) Magnet Recognition, a proof of nursing excellence. This designation was received in July 2022 and placed the hospital in the select group of 15 institutions outside the United States that achieved the Magnet Recognition, being the only one in the Latin America.

### 2.2 Sample

In consonance with the exploratory approach of the project, a convenience sampling strategy was applied. The sample consisted of 652 nurses that occupy an effective nursing position at the bedside care in the institution in July 2020. Those who were on sick leave or away from work during the data collection period were excluded. A total of 168 valid surveys were returned out of 652 distributed, representing a 25.8% response rate.

### 2.3 Survey instrument

A self‐administered electronic survey was created by the research team based on data from a previous qualitative study, in which tactical nurse leaders from the same institution were interviewed and detailed the solutions they designed to fight the pandemic (Freitas et al., 2021). Considering the singularity of the aim of this work, it was not feasible to use a pre‐existing scale.

The survey is composed of 66 solutions, distributed into four categories: (1) physical structure, equipment and materials (16 items); (2) human resources, training, teamwork and communication (19 items); (3) employee, patient and family safety (15 items) and (4) employee comfort (16 items). For each solution, participants were asked to indicate whether they agreed, disagreed or did not know about the solution proposed by the tactical leaders.

### 2.4 Data collection

The contact of the 652 nurses was obtained from the institution's Human Resources department. An official email was sent by the researcher to all target participants, containing a summary of the nature and scope of the study and the link to the survey in Qualtrics Survey Software®.

Participants were informed of the voluntary nature of participation, including the right to withdraw at any time and the right to anonymity. The survey was open for 30 days from 1 July to 31 July 2020, and email reminders were sent to improve the response rate.

### 2.5 Data analysis

Participants' characterization variables were described in absolute frequencies and percentages. Calculations were performed with SPSS software.

### 2.6 Ethical considerations

The study was approved by the National Commission for Research Ethics (Presentation Certificate for Ethical Appreciation number 3.974.416), and all methods were performed in accordance with the Declaration of Helsinki and Brazilian regulations. Electronic informed consent was obtained from all participants.

## 3 RESULTS

A total of 168 nurses participated in this study, most aged between 31 and 40 years (42.9%) with 1.1 to 10 years of experience in the hospital (54.8%). During the pandemic, a part of the nurses was transferred from their work sectors (28.0%), mainly to COVID‐19‐designated units (78.7%). The complete characterization of the sample is presented in Table 1.

**TABLE 1:** Sociodemographic characterization of participants (*n* = 168)

Age (years):	
≤30	36 (21.4%)
31 to 40	72 (42.9%)
41 to 50	51 (30.4%)
≥51	9 (5.4%)
Time since graduation (years):	
≤1	3 (1.8%)
1.1 to 5	37 (22.0%)
5.1 to 10	48 (28.6%)
≥11	80 (47.6%)
Length of experience at the institution (years):	
≤1	38 (22.6%)
1.1 to 5	46 (27.4%)
5.1 to 10	46 (27.4%)
≥11	38 (22.6%)
Were you transferred to another unit during the COVID‐19 pandemic?	
No	121 (72.0%)
Yes	47 (28.0%)
If transferred from another unit, what was your original unit?	
General	35 (74.5%)
Pneumology unit, semi‐ICU, ICU	12 (25.5%)
Total	47 (100.0%)
If transferred from another unit, did you perform your duties in COVID‐19‐designated units?	
No	10 (21.3%)
Yes	37 (78.7%)
Total	47 (100.0%)

The description of the nurses' perception regarding the implementation of solutions proposed by their nurse leaders is presented in Tables 2, 3, 4, 5, according to the category of each solution. In this study, we considered the most effective solutions those that achieved an agreement above 97%, being clearly identified by the bedside nurses. The least effective are those with less than 75% agreement.

**TABLE 2:** Perception of bedside nurses regarding solutions related to physical structure, equipment and materials (*n* = 168)

	Disagree	Agree	Do not know
Level of effectiveness below 75%			
The hospital infection control service performed safety tests on the aprons to assess their quality and safety before they were purchased during the COVID‐19 pandemic.	5 (3.0%)	93 (55.4%)	70 (41.7%)
Level of effectiveness between 75% and 97%			
Negative pressure beds were sufficient to meet the demand of patients during the COVID‐19 pandemic.	28 (16.7%)	132 (78.6%)	8 (4.8%)
The number of equipment purchased to face the COVID‐19 pandemic, such as mechanical ventilators, multiparameter monitors and infusion pumps, was adequate.	1 (0.6%)	160 (95.2%)	7 (4.2%)
The quality of the equipment purchased to face the COVID‐19 pandemic, such as mechanical ventilators, multiparameter monitors and infusion pumps, was adequate.	4 (2.4%)	155 (92.3%)	9 (5.4%)
The hospital adopted the correct respiratory protective masks for employee safety during the COVID‐19 pandemic.	10 (6.0%)	158 (94.0%)	0 (0.0%)
The hospital provided an appropriate and identified place for the storage of the professionals' non‐disposable PPE, such as glasses and N95 masks, during the COVID‐19 pandemic.	9 (5.4%)	158 (94.0%)	1 (0.6%)
In case of inadequacy of any of the PPE used to fight the COVID‐19 pandemic, measures were promptly adopted by the institution to replace it.	3 (1.8%)	149 (88.7%)	16 (9.5%)
The cleaning of environments, equipment, objects and any other materials was carried out with the appropriate frequency and manner during the COVID‐19 pandemic.	5 (3.0%)	162 (96.4%)	1 (0.6%)
The hospital provided a place and adequate supplies for the cleaning of the professionals' non‐disposable PPE, such as glasses and face shields, during the COVID‐19 pandemic.	2 (1.2%)	163 (97.0%)	3 (1.8%)
Safety measures were adopted during the COVID‐19 pandemic on units without negative pressure beds, such as the installation of a door in the corridor for separation of environments and the mandatory use of an N95 mask upon entry into the unit.	4 (2.4%)	163 (97.0%)	1 (0.6%)
The quality of PPE, such as N95 masks, aprons and face shields, available to face the COVID‐19 pandemic, was adequate.	4 (2.4%)	163 (97.0%)	1 (0.6%)
Level of effectiveness above 97%			
The hospital's physical structure was appropriate to face the COVID‐19 pandemic.	4 (2.4%)	164 (97.6%)	0 (0.0%)
The number of medical equipment and supplies acquired to face the COVID‐19 pandemic was adequate.	2 (1.2%)	166 (98.8%)	0 (0.0%)
The quality of the medical equipment and supplies acquired to face the COVID‐19 pandemic was adequate.	1 (0.6%)	166 (98.8%)	1 (0.6%)
The number of PPE, such as N95 masks, aprons and face shields, available to face the COVID‐19 pandemic, was adequate.	3 (1.8%)	164 (97.6%)	1 (0.6%)
Alcohol‐based hand sanitizer was available to employees in adequate quantities and locations during the COVID‐19 pandemic.	1 (0.6%)	167 (99.4%)	0 (0.0%)

Abbreviation: PPE, personal protective equipment.

**TABLE 3:** Perception of bedside nurses regarding human resources, training, teamwork and communication (*n* = 168)

	Disagree	Agree	Do not know
Level of effectiveness below 75%			
The integration of new employees after hiring was done properly during the pandemic.	30 (17.9%)	109 (64.9%)	29 (17.3%)
The training of employees of support areas, such as hospitality, patient transport, nutrition, hygiene, attire and undressing, was carried out in the appropriate time and was sufficient for a safe practice during the COVID‐19 pandemic.	16 (9.5%)	124 (73.8%)	28 (16.7%)
Team meetings with institutional leadership (CEO and directors) took place with adequate frequency during the COVID‐19 pandemic.	8 (4.8%)	124 (73.8%)	36 (21.4%)
Level of effectiveness between 75% and 97%			
The number of employees in your unit was adequate to face the pandemic.	28 (16.7%)	140 (83.3%)	0 (0.0%)
The relocation of employees was carried out timely and adequately to the needs during the COVID‐19 pandemic.	20 (11.9%)	141 (83.9%)	7 (4.2%)
The training of frontline health care workers regarding attire and undressing was carried out at the appropriate time and was sufficient for a safe practice during the COVID‐19 pandemic.	10 (6.0%)	153 (91.1%)	5 (3.0%)
The training of frontline health care workers for the care of patients suspected of or diagnosed with the new coronavirus was carried out at the appropriate time and was sufficient for a safe practice during the COVID‐19 pandemic.	13 (7.7%)	149 (88.7%)	6 (3.6%)
The training of frontline health care workers for the care of critically ill patients was carried out at the appropriate time and was sufficient for a safe practice during the COVID‐19 pandemic.	24 (14.3%)	135 (80.4%)	9 (5.4%)
The training of frontline health care workers in the use of new equipment and supplies was carried out at the appropriate time and was sufficient for safe practice during the COVID‐19 pandemic.	20 (11.9%)	142 (84.5%)	6 (3.6%)
Educational videos and specific educational guidelines were made available during the COVID‐19 pandemic.	7 (4.2%)	160 (95.2%)	1 (0.6%)
Information related with the volume of medical care for patients diagnosed with the new coronavirus, HIAE's installed capacity and demand projections were made available in real time during the COVID‐19 pandemic.	17 (10.1%)	145 (86.3%)	6 (3.6%)
The institution leaders acted proactively during the COVID‐19 pandemic.	8 (4.8%)	159 (94.6%)	1 (0.6%)
Team meetings with respective sectoral leadership took place with adequate frequency during the COVID‐19 pandemic.	20 (11.9%)	143 (85.1%)	5 (3.0%)
You were clear about which leader to report to if your immediate leader was removed for some reason.	7 (4.2%)	159 (94.6%)	2 (1.2%)
Your subordinates were clear about which leader to report to if you were removed for any reason.	5 (3.0%)	159 (94.6%)	4 (2.4%)
There was collaboration between the various areas of the institution during the COVID‐19 pandemic.	6 (3.6%)	162 (96.4%)	0 (0.0%)
The interaction between employees from different areas of the hospital was intense and positive during the COVID‐19 pandemic.	6 (3.6%)	162 (96.4%)	0 (0.0%)
Level of effectiveness above 97%			
There was teamwork during the pandemic.	3 (1.8%)	165 (98.2%)	0 (0.0%)
Employee adherence to PPE was adequate during the COVID‐19 pandemic.	3 (1.8%)	165 (98.2%)	0 (0.0%)

Abbreviations: HIAE, Hospital Israelita Albert Einstein; CEO, Chief Executive Officer; PPE, personal protective equipment.

**TABLE 4:** Perception of bedside nurses regarding the safety of employees, patients, and family members (*n* = 168)

	Disagree	Agree	Do not know
Level of effectiveness below 75%			
The patients' companions were properly attired during the COVID‐19 pandemic.	37 (22.0%)	115 (68.5%)	16 (9.5%)
Level of effectiveness between 75% and 97%			
The flow of medical care for non‐coronavirus patients was communicated and adequately executed during the COVID‐19 pandemic.	6 (3.6%)	160 (95.2%)	2 (1.2%)
The flow of medical care for patients suspected or diagnosed with the new coronavirus was communicated and properly executed during the COVID‐19 pandemic.	5 (3.0%)	163 (97.0%)	0 (0.0%)
Up‐to‐date information and scientific evidence related with the medical care for patients suspected or diagnosed with the new coronavirus were provided to the teams in adequate quantity and time during the COVID‐19 pandemic.	8 (4.8%)	156 (92.9%)	4 (2.4%)
The audit visits to verify the use of PPE and the attire and undressing processes were adequate to promote safe behaviour during the COVID‐19 pandemic.	18 (10.7%)	134 (79.8%)	16 (9.5%)
The suspension of visits to hospitalized patients was carried out properly and timely during the COVID‐19 pandemic.	14 (8.3%)	146 (86.9%)	8 (4.8%)
The stay of a companion in the hospitalized patients' rooms was adequately evaluated by the area coordinators during the COVID‐19 pandemic.	10 (6.0%)	143 (85.1%)	15 (8.9%)
The temperature measurement of employees, patients and companions before entering the hospital was properly performed during the COVID‐19 pandemic.	16 (9.5%)	148 (88.1%)	4 (2.4%)
Patients felt safe in the hospital during the COVID‐19 pandemic.	9 (5.4%)	150 (89.3%)	9 (5.4%)
Patients' companions felt safe in the hospital during the COVID‐19 pandemic.	15 (8.9%)	143 (85.1%)	10 (6.0%)
Employees felt safe in the hospital during the COVID‐19 pandemic.	17 (10.1%)	151 (89.9%)	0 (0.0%)
Patient and employee safety has always been an institutional premise, and it has not been abandoned at any time during the COVID‐19 pandemic.	7 (4.2%)	161 (95.8%)	0 (0.0%)
Level of effectiveness above 97%			
Employees diagnosed with the new coronavirus were promptly removed from work during the COVID‐19 pandemic.	4 (2.4%)	164 (97.6%)	0 (0.0%)
Institutional protocols and guidelines were updated for the prevention of transmission of the new coronavirus during the COVID‐19 pandemic.	3 (1.8%)	165 (98.2%)	0 (0.0%)
Safety guidelines for the care of patients suspected of or diagnosed with the new coronavirus were designed and communicated to the teams during the COVID‐19 pandemic.	1 (0.6%)	166 (98.8%)	1 (0.6%)

Abbreviation: PPE, personal protective equipment.

**TABLE 5:** Perception of bedside nurses regarding solutions for the collaborator's comfort (*n* = 168)

	Disagree	Agree	Do not know
Level of effectiveness below 75%			
The remote work policy during the COVID‐19 pandemic, when applicable, was adequate and promoted comfort.	6 (3.6%)	100 (59.5%)	62 (36.9%)
The provision of hotel accommodations to frontline health care workers during the COVID‐19 pandemic was adequate and promoted comfort.	11 (6.5%)	123 (73.2%)	34 (20.2%)
The support offered to employees' children during the COVID‐19 pandemic, through the maintenance of the day care centre and the partnership with Miguel de Cervantes school, was adequate and promoted comfort.	3 (1.8%)	118 (70.2%)	47 (28.0%)
The dispatch of alcohol‐based hand sanitizer and masks to employees on leave due to diagnosis with the new coronavirus was adequate during the COVID‐19 pandemic and promoted comfort.	5 (3.0%)	110 (65.5%)	53 (31.5%)
Level of effectiveness between 75% and 97%			
The flexibility of working hours during the COVID‐19 pandemic, when applicable, was adequate and promoted comfort.	20 (11.9%)	126 (75.0%)	22 (13.1%)
The adoption of interactivity tools such as Zoom during the COVID‐19 pandemic was adequate and promoted comfort.	5 (3.0%)	147 (87.5%)	16 (9.5%)
The hospitalization and treatment of collaborators diagnosed with the new coronavirus at HIAE during the COVID‐19 pandemic was adequate and provided comfort.	2 (1.2%)	158 (94.0%)	8 (4.8%)
The expansion of the service hours of occupational care clinic during the COVID‐19 pandemic was adequate and promoted comfort.	7 (4.2%)	142 (84.5%)	19 (11.3%)
The expansion of medical care via the telemedicine program during the COVID‐19 pandemic was adequate and provided comfort.	7 (4.2%)	147 (87.5%)	14 (8.3%)
The psychological support of the ‘POP’ program during the COVID‐19 pandemic was adequate and provided comfort.	9 (5.4%)	127 (75.6%)	32 (19.0%)
The implementation of the ‘OUVID ‐ Listening is also caring’ program during the COVID‐19 pandemic was adequate and promoted comfort.	10 (6.0%)	142 (84.5%)	16 (9.5%)
The availability of the Carrefour Express minimarket and the Swift Meat Market truck on the hospital facilities during the COVID‐19 pandemic was adequate and promoted comfort.	2 (1.2%)	163 (97.0%)	3 (1.8%)
The provision of nap cabins on the hospital premises during the COVID‐19 pandemic was adequate and promoted comfort.	23 (13.7%)	128 (76.2%)	17 (10.1%)
The availability of decompression areas (quiet spaces with sofas, chairs and side tables) during the COVID‐19 pandemic was adequate and promoted comfort.	19 (11.3%)	131 (78.0%)	18 (10.7%)
The offer of balanced and reinforced snacks to frontline health care workers, including fruits, salty snacks and juices, during the COVID‐19 pandemic, was adequate and promoted comfort.	11 (6.5%)	128 (76.2%)	29 (17.3%)
The employee safety and comfort measures adopted by the institution were important to reduce their stress during the COVID‐19 pandemic.	12 (7.1%)	150 (89.3%)	6 (3.6%)

Abbreviation: PPE, personal protective equipment.

The first category evaluated solutions related to physical structure, equipment and materials (Table 2) and was the one with the highest degree of agreement among the four evaluated, which means that bedside nurses were able to easily recognize these solutions in their practice. The solution ‘Alcohol‐based hand sanitizer was available to employees in adequate quantities and locations during the COVID‐19 pandemic’ achieved 99.4% of agreement. On the other hand, the solution ‘The Hospital Infection Control Service performed safety tests on the aprons to assess their quality and safety before they were purchased during the COVID‐19 pandemic’ seems to be unknown by bedside nurses (41.7%).

In the second category, solutions associated to human resources, training, teamwork and communication were assessed (Table 3). The bedside nurses agreed that ‘There was teamwork during the pandemic’ (98.2%). However, only 64.9% agreed that ‘The integration of new employees after hiring was done properly during the pandemic’.

The third category addressed solutions related to employee, patient and family safety (Table 4). More than 98% of bedside nurses recognized that ‘Institutional protocols and guidelines were updated for the prevention of transmission of the new coronavirus during the COVID‐19 pandemic’ and ‘Safety guidelines for the care of patients suspected of or diagnosed with the new coronavirus were designed and communicated to the teams during the COVID‐19 pandemic’. Nevertheless, 22% of bedside nurses disagreed that ‘The patients' companions were properly attired during the COVID‐19 pandemic’.

The last category evaluated solutions belonging to employee comfort (Table 5). In general, this category reached the lowest levels of agreement. The only solutions that reached more than 90% agreement were ‘The hospitalization and treatment of collaborators diagnosed with the new coronavirus at HIAE during the COVID‐19 pandemic was adequate and provided comfort’ and ‘The availability of the Carrefour Express minimarket and the Swift Meat Market truck on the hospital facilities during the COVID‐19 pandemic was adequate and promoted comfort’. A significant portion of the participants (31.5%) did not know about ‘The dispatch of alcohol‐based hand sanitizer and masks to employees on leave due to diagnosis with the new coronavirus’.

## 4 DISCUSSION

The present study identified important aspects of the implementation of solutions for patient and employee care during the COVID‐19 pandemic in a Brazilian hospital considered the national reference for the novel coronavirus.

Because of the unprecedented situation, nurse leaders were forced to adopt unexampled measures to manage the nursing workforce ensuring the best care for patients while preventing physical and psychological health problems of nurses. In this survey, bedside nurses recognized most of the solutions proposed by tactical leaders, indicating their effectiveness.

We found most of the nurses are less than 40 years old and used to work in general wards, denoting limited experiences regarding the infectious pandemic and critical care, which is consistent with recent research (Cai et al., 2020; Cotrin et al., 2020; Santos et al., 2021).

Rotation of nurses from one unit to another was a measure widely adopted at the beginning of the pandemic to cope with the growing number of COVID‐19 patients. In the hospital studied, 28% of nurses were relocated to sectors different than the ones they used to work in, and the majority (78.7%) were transferred to COVID‐19‐designated units. In addition to the relocation of nurses, some hospitals also attempted to merge more experienced nurses with newly admitted professionals into the work environment and leave risk groups out of situations where the risk of contamination was high (Gagliano et al., 2020; Poortaghi et al., 2021).

Research conducted in Wuhan evaluated the rotation of medical staff (surgeons and nurses) to provide support in the work against COVID‐19 and demonstrated that after adequate training and with appropriate precautions, it was safe and effective (Wen et al., 2021).

Four categories of solutions designed to fight the pandemic were assessed in our study: physical structure, equipment and materials; human resources, training, teamwork and communication; employee, patient and family safety and employee comfort.

Among the solutions related to the physical structure, equipment and materials, the ones most perceived by the bedside nurses were the strategies for structure adequacy, adaptation of beds to receive COVID‐19 patients, acquisition of medical equipment and supplies and availability of alcohol‐based hand sanitizer and personal protective equipment (PPE). Conversely, almost 42% of participants reported not knowing about the safety testing on aprons prior to purchasing, even though they have been performed.

The nature of the actions can explain this divergence. The adaptation of beds and the availability of supplies can be more visible to those in care than internal and undisclosed processes, such as material safety testing prior to acquisition conducted by professionals from the Hospital Infection Control Service.

Nurses' perception of solutions for the adequacy of human resources and training, teamwork and adherence to the use of PPE stood out positively. A relevant and frequent topic in discussions and publications about COVID‐19 was the effort of the bedside teams to carry out effective teamwork. Strategies used to improve teamwork include trust in leadership, transparent and shared decision‐making, continuous communication, patient focus and mutual support among team members, which should remain after the end of the pandemic (Jassar et al., 2021; Natale et al., 2020).

Other solutions in the human resources, training, teamwork and communication category showed high levels of disagreement and unfamiliarity, such as training of employees in support areas for the correct use of PPE, team meetings with institutional leaders (CEO and directors) and the integration of newly hired employees. The intensity of the work, the uniqueness of the situations experienced and the great involvement in the team tasks may justify the nurses' unawareness of these solutions.

Regarding the reception and support to temporary and newly admitted nurses, the Australian Nursing and Midwifery Federation published a statement highlighting the need for integration into the institution, with all the necessary training and provision of institutional protocols, focusing on the new member's attributions and safeguarding their safety (Peters, 2020).

Ensuring the safety of all employees, patients and family members was a challenge, requiring constant adaptation and updating of organizational guidelines and workflows, and this was widely recognized by study participants. Research carried out with bedside nurses from several US hospitals support the fundamental role of the structural adjustment and adaptation of care processes for quality and safe care in the pandemic scenario (Schroeder et al., 2020). The dissemination, agreement and adherence to the guidelines and workflows established to face the pandemic may have been specifically facilitated in the studied institution as it is aligned with the most important global health recommendations, evidenced by its several international certifications.

A safety‐related item of greatest disagreement and unfamiliarity was the attire of the patient's companions, despite the fact that they had aprons, gloves and masks readily available throughout the hospital units. This might be explained by the difficulty in the recognition by the patient's companions of the importance of the PPE's correct use, their technical inability and the absence of specific professionals to provide guidance (Estequi, 2021).

Supporting employees who are either healthy or sick with COVID‐19 and promoting their comfort was also a challenge. Several authors recommend institutional support but do not define specific guidelines (Duprat & Melo, 2020; Wang et al., 2020). The studied hospital already had a well‐established employee health and wellness program, which was expanded and strengthened during the pandemic.

The availability of convenience stores and services within the institution was a solution widely recognized as promoting comfort. Restrictions on the movement of people and the operation of stores and services may explain this finding, as they limited access to essential services and goods. Other comfort solutions were unfamiliar to many bedside nurses, including the policy of remote work, provision of masks and hand sanitizer for sick employees isolated at home, hotel accommodations for frontline health care workers to avoid contact with family members and day care centre for children so that they would be safe while their parents were working at the hospital.

Recommendations to promote employee comfort found in the literature include providing areas for rest and physical activity, offering psychological and psychiatric support, establishing partnerships with food companies and providing schools for workers' children (Cho et al., 2021; Howell, 2021; Wang et al., 2020).

Reflecting on all the points discussed in the study, we can visualize that the response to a challenge as big as the COVID‐19 pandemic required complex and quick solutions, which involved numerous processes, flows, resources and, especially, many nursing workers, ‘so human’ as the patients who need their care.

Thus, the most significant learning reminds us of an old assumption in health that the pandemic brought up: caring (in the broadest sense) first of those who care, so that they can offer their care in the form of work, in the most qualified way possible.

There are some limitations in our study. First, because of the pandemic, there was a possible bias in the participation of nurses, as we used online research. Second, the research evaluated the nurses' perception, which is susceptible to a series of cognitive, perceptual and motivational biases that may have led to higher or lower rates of agreement for each solution evaluated. Third, we did not qualitatively assess nurses' perceptions to find explanations for agreement rates.

The findings of this study can contribute to strengthening the response to the COVID‐19 pandemic, whose end is still very uncertain, and be a source of learning and development for new outbreaks such as Monkeypox, declared in July 2022 a public health emergency of international concern by the WHO.

## 5 CONCLUSIONS

Bedside nurses were able to recognize most of the solutions proposed by their nurse leaders for patient and employee care during the COVID‐19 pandemic, highlighting those related to adaptations of the physical structure, availability of medical supplies and adequacy of institutional protocols, guidelines and workflows.

On the other hand, important solutions were more unfamiliar to bedside nurses, such as the adequate integration of new employees and the availability of remote work, hotel accommodations for frontline health care workers and day care for children whose parents worked at the hospital.

This study highlights the possibility of verifying how actions determined at the tactical level affect bedside nurses, which is a constant challenge for leadership. It is of great importance to carry out research that consider operational aspects of tactical decisions, favouring the precise targeting of strategies capable to respond to the needs of everyone involved.

## 6 IMPLICATIONS FOR NURSING MANAGEMENT

Our research showed that tactical‐level nurse leaders need constant proximity to bedside nurses and continuous elucidation of the objectives to be achieved by the strategies adopted.

Tactical nurse leaders need to communicate the results that are expected of those they lead. However, especially in adverse times, it is essential to be clear while disseminating and implementing measures directed to personal needs, as they can generate a sense of understanding and comfort.

At a time as critical as the COVID‐19 pandemic, if bedside nurses are focused on their work performance, tactical nurses must broaden their view and point out also aim for a favourable work environment, safety and comfort of the nursing team.

## CONFLICTS OF INTEREST

The authors declare no conflicts of interest.

## ETHICAL APPROVAL

The study was approved by the National Research Ethics Committee–CONEP (Presentation Certificate for Ethical Appreciation number 3.974.416), and all methods were performed in accordance with the Declaration of Helsinki and Brazilian regulations. Electronic informed consent was obtained from all participants.

## Data Availability

The data that support the findings of this study are available on request from the corresponding author. The data are not publicly available because of privacy or ethical restrictions.
